# Preparation of a chronic complete atrioventricular block rat model using myocardial surface radiofrequency ablation

**DOI:** 10.3389/fphar.2026.1900919

**Published:** 2026-08-28

**Authors:** Ying Wang, Shaojun Xu, Juan Wang, Le Zhao, Jinyan Zhang

**Affiliations:** Beijing Key Laboratory of Pharmacology of Chinese Materia Medic, Institute of Basic Medical Sciences of Xiyuan Hospital, China Academy of Chinese Medical Sciences, Beijing, China

**Keywords:** chemical ablation, complete atrioventricular block, model, radiofrequency ablation, rat

## Abstract

Research using rat models of chronic complete atrioventricular block (CAVB) remains limited, and several problems, particularly model instability, persist. This study aimed to optimize the method used to establish a CAVB rat model. The most suitable approach for establishing a CAVB rat model was selected from chemical ablation, myocardial invasive radiofrequency ablation, and myocardial surface radiofrequency ablation by comparing their success rates and stability. The findings indicate that myocardial surface radiofrequency ablation using a thin blunt tip is a reliable method for establishing a CAVB rat model, offering a high success rate, operational simplicity, and good stability. Moreover, myocardial surface radiofrequency ablation with a thin blunt tip achieved comparable success rates under two different parameter settings (20 W/5 s and 25 W/3 s). An appropriately sized contact tip should be selected to prevent accidental contact with the aorta. The ablation parameters can be adjusted according to the principle that lower power requires a longer ablation duration, whereas higher power permits a shorter duration. This CAVB rat model is important for further investigating the mechanisms underlying CAVB and developing more effective therapeutic interventions.

## Graphical Abstract

**Figure:**
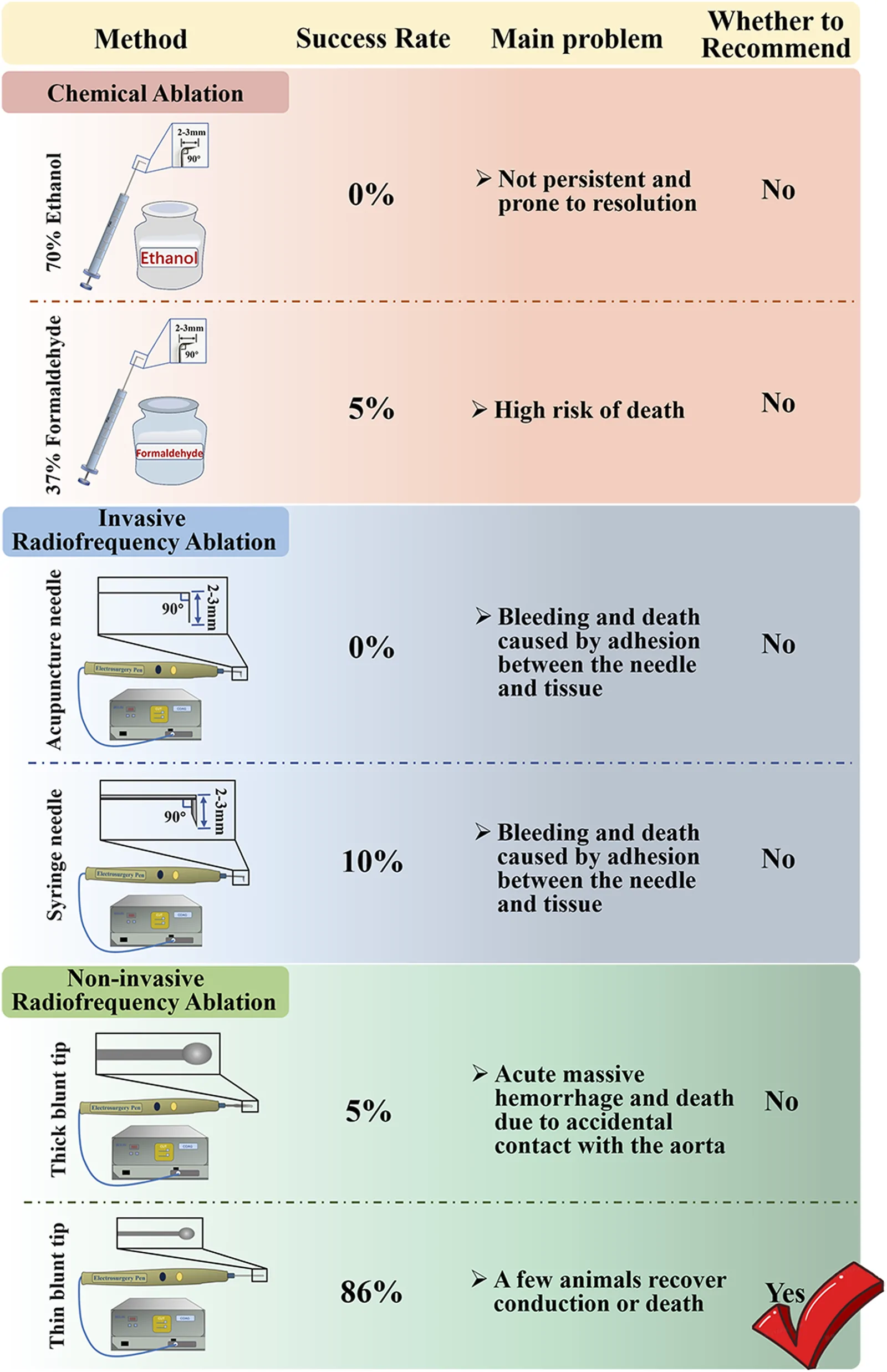

## 1 Introduction

Complete atrioventricular block (CAVB), also referred to as third-degree atrioventricular block, is characterized by the complete interruption of electrical impulse conduction from the atria to the ventricles. Its clinical manifestations include fatigue, dizziness, palpitations, reduced exercise capacity, and generalized weakness, which may progress to syncope or sudden death in severe cases. Current treatment primarily involves implantation of an electronic cardiac pacemaker, which can improve quality of life to some extent. However, the high cost of pacemaker implantation, which ranges from $2500 to $10,000 (Joury et al., 2021), together with the requirement for regular device monitoring and maintenance, places a substantial financial burden on patients and their families. Moreover, pacemaker implantation may not be advisable for growing children, elderly individuals with comorbidities, pregnant women, or patients with certain conditions. Pacemaker implantation also does not provide a permanent solution because complications such as lead failure and battery depletion may occur (Tjong and Reddy, 2017). Further investigation of the mechanisms underlying CAVB and the development of more effective therapeutic approaches are therefore essential. Establishment of a reliable animal model of CAVB is necessary for investigating its pathogenesis and developing appropriate treatment strategies.

At present, most CAVB models have been established in large animals, including dogs, pigs, and sheep, through radiofrequency ablation under open- or closed-chest conditions (Loen et al., 2022; Lehmann et al., 2017; Smoczyńska et al., 2020; Huang et al., 1987; Bru et al., 2002; Sill et al., 2011). Open-chest procedures require prior pacemaker implantation to ensure survival, whereas closed-chest procedures depend on fluoroscopic guidance. Consequently, establishing CAVB models in large animals is difficult under conventional laboratory conditions because these procedures are complex, technically demanding, expensive, and resource-intensive. Rodents, particularly rats, are extensively used in laboratory experiments because of their small size and low cost. Nevertheless, few studies have examined CAVB models in rats over the past 50 years (Table 2). Previous studies have frequently reported high animal mortality, model instability, and short maintenance periods, which limit reproducibility and broader application. Therefore, this study aimed to optimize the existing methods and establish a simple, highly successful, and stable CAVB rat model.

## 2 Materials and methods

### 2.1 Reagents and materials

Absolute ethanol and 37% formaldehyde were utilized. All reagents were of analytical grade. An acupuncture needle (0.3 × 50 mm, Beijing Zhongyan Taihe Medical Instrument Co., Ltd.), a disposable medical syringe (1 mL, Jiangxi Fenglin Medical Instrument Co., Ltd.), and microliter syringes (100 μL, Shanghai Gaoge Industry and Trade Co., Ltd.) were used.

### 2.2 Instruments

An ALC-V8S small-animal ventilator (Shanghai Alcott Biotech Co., Ltd.), a DGD-300C-2 medical computerized high-frequency electrotome (Beijing Beilin Electronics Co., Ltd.), an electrocardiogram (ECG) acquisition and analysis system (Beijing Soft Long Biotechnology Co., Ltd.), and a Vevo 2100 echocardiography system (VisualSonics Inc., Toronto, Canada) were employed.

### 2.3 Animals

**FIGURE 1:**
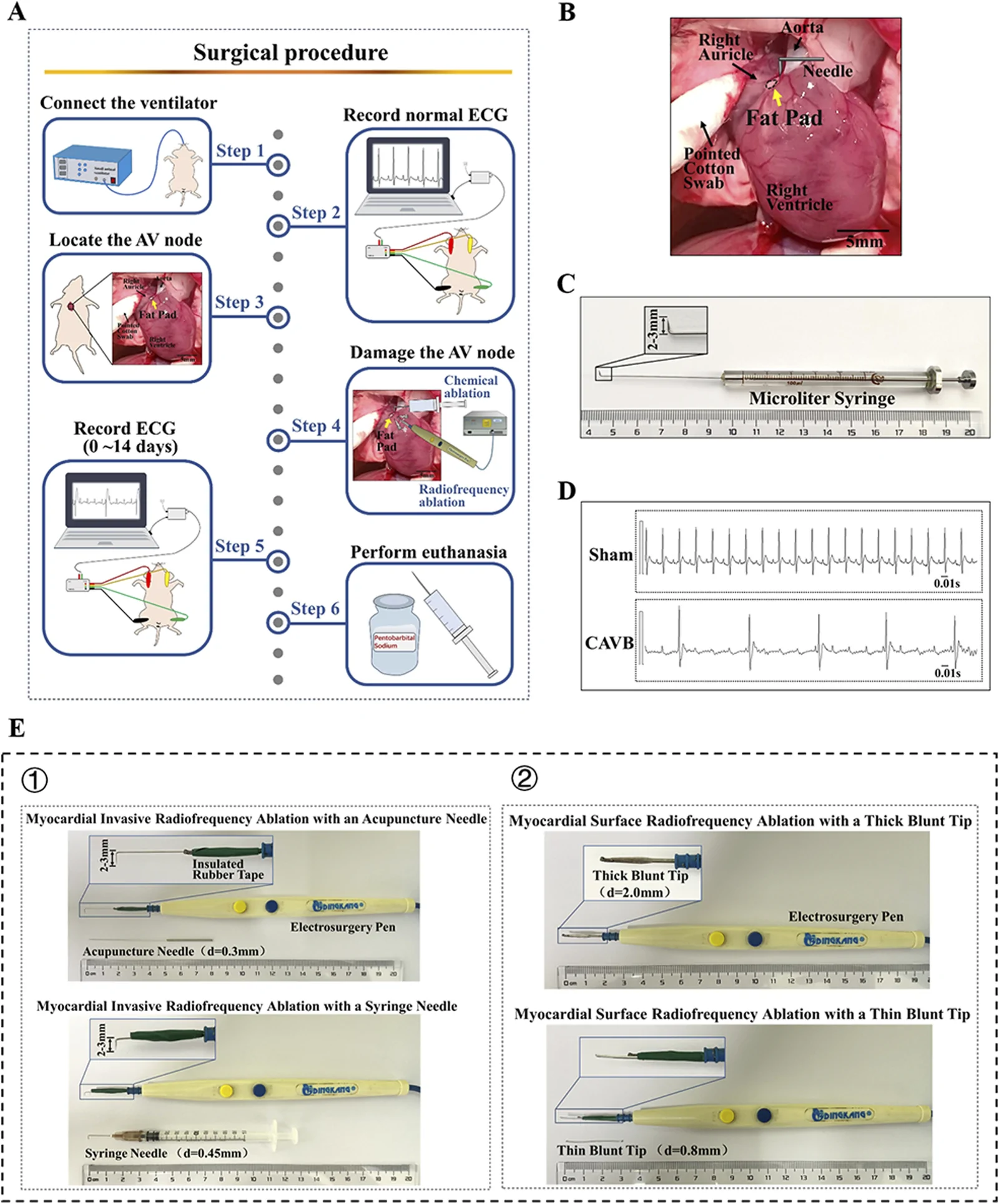
Experimental procedures for establishing a rat model of complete atrioventricular block (CAVB). **(A)** Surgical procedure. **(B)** The atrioventricular (AV) node was localized using the fat pad. **(C)** Chemical ablation device. **(D)** Characteristic electrocardiogram (ECG) of CAVB observed during the experiment, showing a ventricular escape rhythm. **(E)** Devices used for myocardial invasive (①) and myocardial surface (②) radiofrequency ablation.

Healthy adult male Sprague‒Dawley rats weighing 200 ± 25 g were used to establish the CAVB model (Figure 1A). All animal procedures were performed in accordance with Directive 2010/63/EU of the European Parliament on the protection of animals used for scientific purposes or the National Institutes of Health (NIH) Guide for the Care and Use of Laboratory Animals and were approved by the Medical Ethics Committee of Xiyuan Hospital, China Academy of Chinese Medical Sciences (2024XLC093-2). All animal experiments were conducted in strict accordance with the 3R principles. During the operation, if the rats did not exhibit the characteristic ECG features of CAVB, the procedure was repeated no more than twice. Rats that failed to regain consciousness after anesthesia were euthanized with a high dose of anesthetic in accordance with the ethical requirements.

### 2.4 Surgical procedure

#### 2.4.1 Preoperative preparation

The SD rats were anesthetized by intraperitoneal administration of sodium pentobarbital (40 mg/kg) and subsequently underwent endotracheal intubation. The rats were connected to a small-animal ventilator to support respiration, and the respiratory rate and tidal volume were adjusted according to body weight. Following previously described procedures (Ali et al., 2020; Kim et al., 2019), a right-sided thoracotomy was performed between the third and fourth ribs to expose the heart. Before surgery, carprofen (5 mg/kg) was administered subcutaneously, and buprenorphine SR (1 mg/kg) was given for analgesia. All surgical procedures were performed by the same operator. Standard lead II ECG recordings of the rat limbs were obtained before modeling.

#### 2.4.2 Localization of the atrioventricular node

Precise localization and stimulation of the atrioventricular (AV) node to induce injury required complete exposure of the fat pad situated between the medial aspect of the right auricle and the aortic root (Figure 1B). However, the right auricle frequently obstructed the view of the fat pad, making access to the target area difficult. To address this problem, three techniques for stabilizing the right auricle during the procedure were evaluated: (1) clamping the right auricle with surgical forceps, (2) securing it with surgical sutures, and (3) gently retracting it with sterile pointed cotton swabs (Figure 1B). These techniques were comparatively evaluated, after which sterile pointed cotton swabs were selected for use during model establishment.

### 2.5 Chemical ablation

The needle was maintained parallel to the ascending aorta throughout the procedure. The epicardial surface was penetrated at a point 1 mm posterior and 1 mm lateral to the fat pad. The needle was advanced toward the cardiac apex along the long axis of the heart. Insertion of the needle 2–3 mm into the tissue allowed the AV node in the rat heart to be targeted. To control the insertion depth, the microinjector needle was bent by 90° approximately 2–3 mm from its tip. The designated reagent was then injected at the dose specified for each group (Table 1), and subsequent ECG changes were monitored. Each reagent was administered within 10 s. If the ECG features of CAVB were not observed within 1 min, the same reagent dose was administered again, with no more than two attempts.

**TABLE 1:** Parameters of each modeling method.

Method	Reagent/operating tools	Dose/Power		
Chemical ablation	70% ethanol	50 μL	100 μL	​
	37% formaldehyde	30 μL	50 μL	​
Invasive radiofrequency ablation	Acupuncture needles	0 W/5 s	20 W/5 s	25 W/3 s
	1 mL syringe needle	0 W/5 s	20 W/5 s	25 W/3 s
Surface radiofrequency ablation	Thick blunt tip	0 W/5 s	20 W/5 s	25 W/3 s
	Thin blunt tip	0 W/5 s	20 W/5 s	25 W/3 s

#### 2.5.1 Ethanol ablation

Using a random number table, the rats were randomly allocated to three groups of 10 rats each: ① 50 μL of ethanol (70%), ② 100 μL of ethanol (70%), and ③ 100 μL of saline.

#### 2.5.2 Formaldehyde ablation

Using a random number table, the rats were randomly allocated to three groups of 10 rats each: ① 30 μL of formaldehyde (37%), ② 50 μL of formaldehyde (37%), and ③ 50 μL of saline.

### 2.6 Invasive radiofrequency ablation

Insulating rubber tape was used to attach either an acupuncture needle or a 1 mL syringe needle to the blunt tip of a surgical knife. The acupuncture needle or 1 mL syringe needle was bent at a 90° angle approximately 2–3 mm from the tip (Figure 1E) before use. Thoracotomy was performed in the rats according to the procedure described in section “2.4.”. A reusable negative electrode plate was placed on the back of each rat as the return electrode. The surgical knife was operated in coagulation mode, and the AV node tissue was ablated according to the power and duration specified for each group (Table 1). ECG changes after stimulation were subsequently monitored. If the characteristic ECG features were not observed within 1 min, the same stimulation procedure was repeated. No more than two attempts were performed.

#### 2.6.1 Myocardial invasive radiofrequency ablation using an acupuncture needle

Using a random number table, the rats were randomly allocated to three groups of 10 rats each. ① the acupuncture needle (0 W/5 s) group, ② the acupuncture needle (20 W/5 s) group, and ③ the acupuncture needle (25 W/3 s) group.

#### 2.6.2 Myocardial invasive radiofrequency ablation using a syringe needle

Using a random number table, the rats were randomly divided into three groups of 10 rats each: ① the syringe needle (0 W/5 s) group, ② the syringe needle (20 W/5 s) group, and ③ the syringe needle (25 W/3 s) group.

### 2.7 Surface radiofrequency ablation

The thick blunt tip consisted of a 3 cm-long iron wire with a diameter of 2.0 mm, whereas the thin blunt tip consisted of a 3 cm-long iron wire with a diameter of 0.8 mm. One end of each wire, which directly contacted the myocardial tissue, was polished to obtain a smooth surface (Figure 1E). As described in section “2.6.”, the blunt tip was attached to the thick blunt end of the electric knife pen without bending the tip. During stimulation, the tip was gently placed on the myocardial surface between the fat pad and the aorta without penetrating the tissue. The AV node tissue was ablated using the power and duration specified for each group (Table 1). Changes in the ECG after stimulation were then monitored. If the characteristic ECG features were not observed within 1 min, the same stimulation procedure was repeated. The procedure was attempted no more than twice.

#### 2.7.1 Myocardial surface radiofrequency ablation using a thick blunt tip

Using a random number table, the rats were randomly allocated to three groups of 10 rats each: ① the thick blunt tip (0 W/5 s) group, ② the thick blunt tip (20 W/5 s) group, and ③ the thick blunt tip (25 W/3 s) group.

#### 2.7.2 Myocardial surface radiofrequency ablation using a thin blunt tip

Using a random number table, the rats were randomly assigned to the following groups, with 10–15 rats in each group: ① the thin blunt tip (0 W/5 s) group, ② the thin blunt tip (20 W/5 s) group, and ③ the thin blunt tip (25 W/3 s) group.

### 2.8 Model success criteria

The characteristic ECG features of CAVB included an irregular P-R interval caused by the absence of a fixed relationship between the P wave and QRS complex, a fixed and prolonged R-R interval, and two or more consecutive P waves. A model was considered successful when the rats exhibited sustained characteristic ECG features of CAVB (Figure 1D), indicating complete interruption of atrioventricular conduction and the occurrence of a ventricular escape rhythm. In rats subjected to chemical ablation, invasive myocardial radiofrequency ablation, and myocardial surface radiofrequency ablation using a thick blunt tip, ECGs were recorded immediately (0 h) and at 0.5 h, 1 h, 2 h, 24 h, 48 h, 7 days, and 14 days after modeling. In rats subjected to myocardial surface radiofrequency ablation using a thin blunt tip, ECGs were recorded immediately (0 h) and at 1, 2, and 4 weeks after modeling. During ECG acquisition, the rats were anesthetized with 2% isoflurane. ECGs were continuously recorded for 5 min at each time point, and rats in which characteristic CAVB features were detected during this period were considered to have maintained the CAVB model.

### 2.9 Postoperative care

After model establishment, the muscles and skin of each rat were carefully closed using (3–0) surgical sutures. The rats were placed on a heating pad maintained at 37 °C until consciousness was regained. After awakening, the rats were transferred to a carton maintained at an ambient temperature of 37 °C–39 °C for 1–2 h and were provided free access to food and water. To prevent infection, each rat received a daily intraperitoneal injection of 80,000 units of penicillin for 5 days after surgery.

### 2.10 Echocardiography

Cardiac function after radiofrequency ablation using the thin blunt tip was evaluated at week 4 after surgery with a Vevo 2100 echocardiography system. After anesthesia with 2% isoflurane, the following parameters were measured: left ventricular anterior wall thickness at end-systole and end-diastole (LVAWs and LVAWd), left ventricular posterior wall thickness at end-systole and end-diastole (LVPWs and LVPWd), left ventricular internal diameter at end-systole and end-diastole (LVIDs and LVIDd), and left ventricular end-systolic and end-diastolic volumes (LVESV and LVEDV). Left ventricular fractional shortening (FS), ejection fraction (EF), cardiac output (CO), and stroke volume (SV) were also determined.

### 2.11 Pathological staining

At week 4 after surgery, the rats were euthanized by an overdose of sodium pentobarbital (100 mg/kg, i. p.) after cardiac function data had been collected. The hearts were subsequently excised, fixed in 4% paraformaldehyde, embedded in paraffin, and sectioned. Histopathological changes and collagen deposition in the AVN region were evaluated using H&E and Masson staining, respectively.

### 2.12 Statistical analysis

Data were analyzed using SPSS statistical software (SPSS 26.0, IBM, Armonk, NY), and measurement data are expressed as the mean ± standard deviation (SD). All histograms were generated using GraphPad Prism 9.0 software (GraphPad Software, San Diego, USA). The normality of the distribution was assessed for all variables. For the ECG results, normally distributed data were analyzed using a paired-samples t-test, whereas non-normally distributed data were analyzed using the Wilcoxon signed-rank test. For normally distributed data, one-way ANOVA followed by Tukey’s honestly significant difference (HSD) *post hoc* test or Tamhane’s *post hoc* test was performed. For non-normally distributed data, the Kruskal–Wallis test was used. Pearson’s chi-squared test was applied to compare rates among groups. A *P* value <0.05 was considered statistically significant.

## 3 Results

### 3.1 Chemical ablation produced a very low success rate in establishing the CAVB rat model

#### 3.1.1 Ethanol ablation induced transient and reversible ECG characteristics of CAVB

**FIGURE 2:**
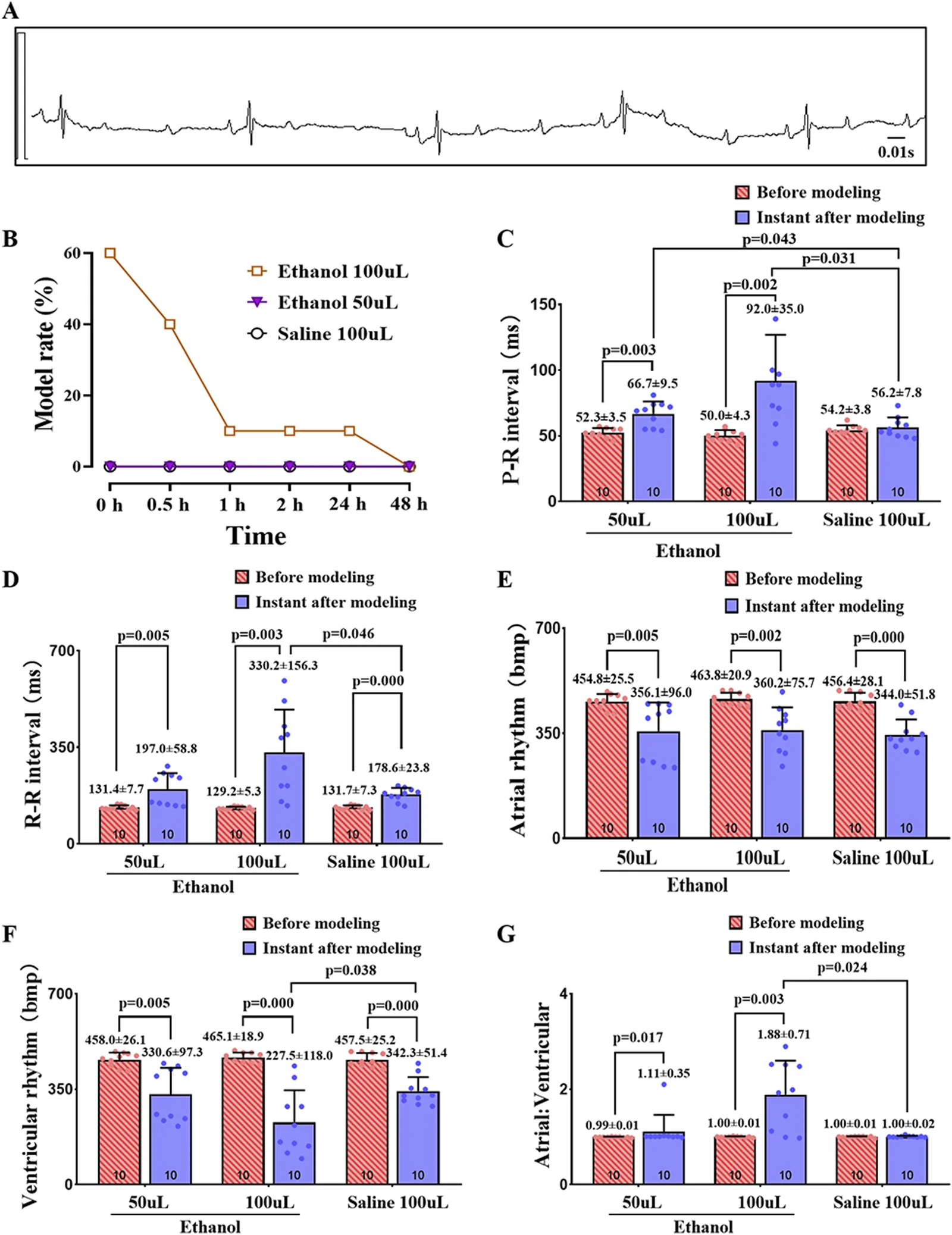
Electrocardiogram (ECG) findings after ethanol ablation of the atrioventricular node in rats (n = 10). **(A)** Characteristic complete atrioventricular block (CAVB) ECG in the 100 µL ethanol group. **(B)** Proportion of rats exhibiting CAVB ECG features. **(C)** P-R interval. **(D)** R-R interval. **(E)** Atrial rhythm. **(F)** Ventricular rhythm. **(G)** Atrial:Ventricular (A:V) beat ratio. Note: The model rate (%) was calculated as follows: (the number of rats exhibiting characteristic CAVB ECG features/the total number of rats) ×100%. All values are presented as the mean ± SD. Paired-samples t-tests or Wilcoxon signed-rank tests was used to assess significant differences in the ECGs results before and after modeling within the same group of rats. One-way ANOVA followed by Tukey’s honestly significant difference (HSD) *post hoc* test and Tamhane’s *post hoc* test or Kruskal–Wallis test was used to evaluate significant differences among groups at the same time point.

Before modeling, all groups exhibited normal ECGs with sustained sinus rhythm (*P* > 0.05). In the 100 µL saline group, the injection of saline into the AV node tissue did not produce characteristic ECG features of CAVB. Although the R-R interval was significantly prolonged (*P* < 0.01), it returned to normal within minutes. In the 50 µL ethanol group, none of the rats initially exhibited characteristic CAVB ECG features after ablation, although both the P-R and R-R intervals were significantly prolonged compared with their pre-ablation values (*P* < 0.01). In the 100 µL ethanol group, the P-R interval became irregular and dissociated, the R-R interval was significantly prolonged, and the atrial-to-ventricular (A:V) beat ratio was significantly increased relative to that before modeling (*P* < 0.01). Ventricular escape rhythm and other ECG features characteristic of CAVB were observed in 60% (6/10) of the rats. Among these six rats, the CAVB ECG features disappeared in four rats within 48 h after ablation, whereas the other two rats died within the same period (Figure 2).

#### 3.1.2 Formaldehyde ablation was associated with high mortality in rats

**FIGURE 3:**
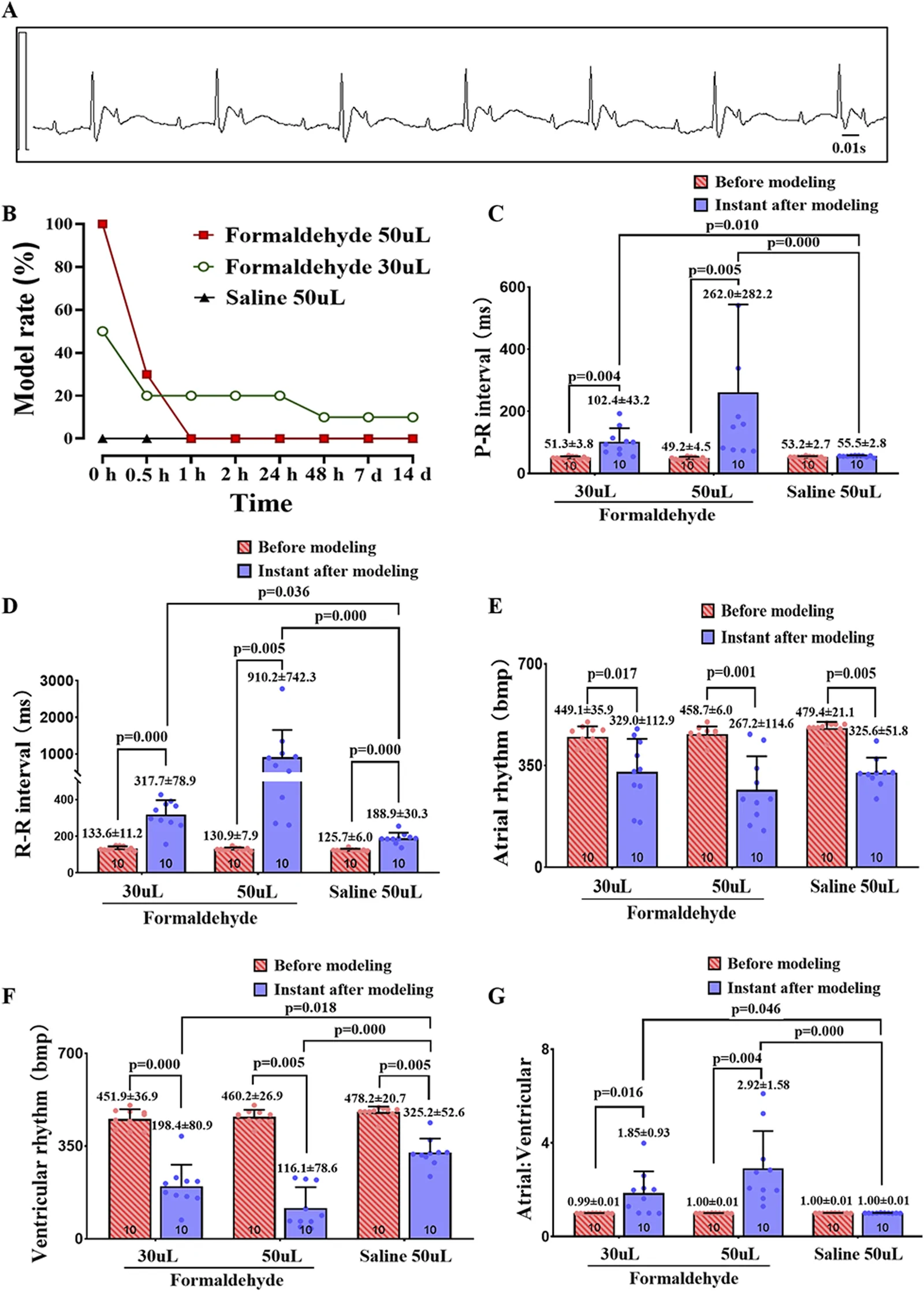
Electrocardiogram (ECG) findings after formaldehyde ablation of the atrioventricular node in rats (n = 10). **(A)** Characteristic complete atrioventricular block (CAVB) ECG showing a ventricular escape rhythm. **(B)** Proportion of rats exhibiting CAVB ECG features. **(C)** P-R interval. **(D)** R-R interval. **(E)** Atrial rhythm. **(F)** Ventricular rhythm. **(G)** Atrial:Ventricular (A:V) beat ratio. Note: The model rate (%) was calculated as follows: (the number of rats exhibiting characteristic CAVB ECG features/the total number of rats) ×100%. All values are presented as the mean ± SD. Paired-samples t-tests or Wilcoxon signed-rank tests was used to assess significant differences in the ECGs results before and after modeling within the same group of rats. One-way ANOVA followed by Tukey’s honestly significant difference (HSD) *post hoc* test and Tamhane’s *post hoc* test or Kruskal–Wallis test was used to evaluate significant differences among groups at the same time point.

Before modeling, all groups exhibited normal ECGs with sustained sinus rhythm (*P* > 0.05). In the 50 µL saline group, the R-R interval was significantly prolonged after saline injection into the AV node tissue (*P* < 0.01) but returned to normal within minutes, and no characteristic CAVB ECG features were observed. After ablation, characteristic CAVB ECG features were detected in 50% (5/10) and 100% (10/10) of the rats in the 30 μL and 50 µL formaldehyde groups, respectively. In both groups, the P-R interval was irregular and dissociated, the R-R interval was significantly prolonged and the atrial-to-ventricular (A:V) beat ratio was significantly increased compared with that before modeling (*P* < 0.05, *P* < 0.01). Among the 15 rats exhibiting CAVB ECG features, only 1 rat in the 30 µL formaldehyde group maintained these features for more than 14 days. Of the remaining rats, 13 died within 1 h after ablation, and 1 no longer exhibited the ECG features of CAVB within 48 h after ablation (Figure 3).

### 3.2 Myocardial invasive radiofrequency ablation had a very low success rate in establishing the CAVB rat model

#### 3.2.1 The acupuncture needle rarely induced CAVB ECG features and readily caused bleeding and death because of needle adhesion to the tissue

**FIGURE 4:**
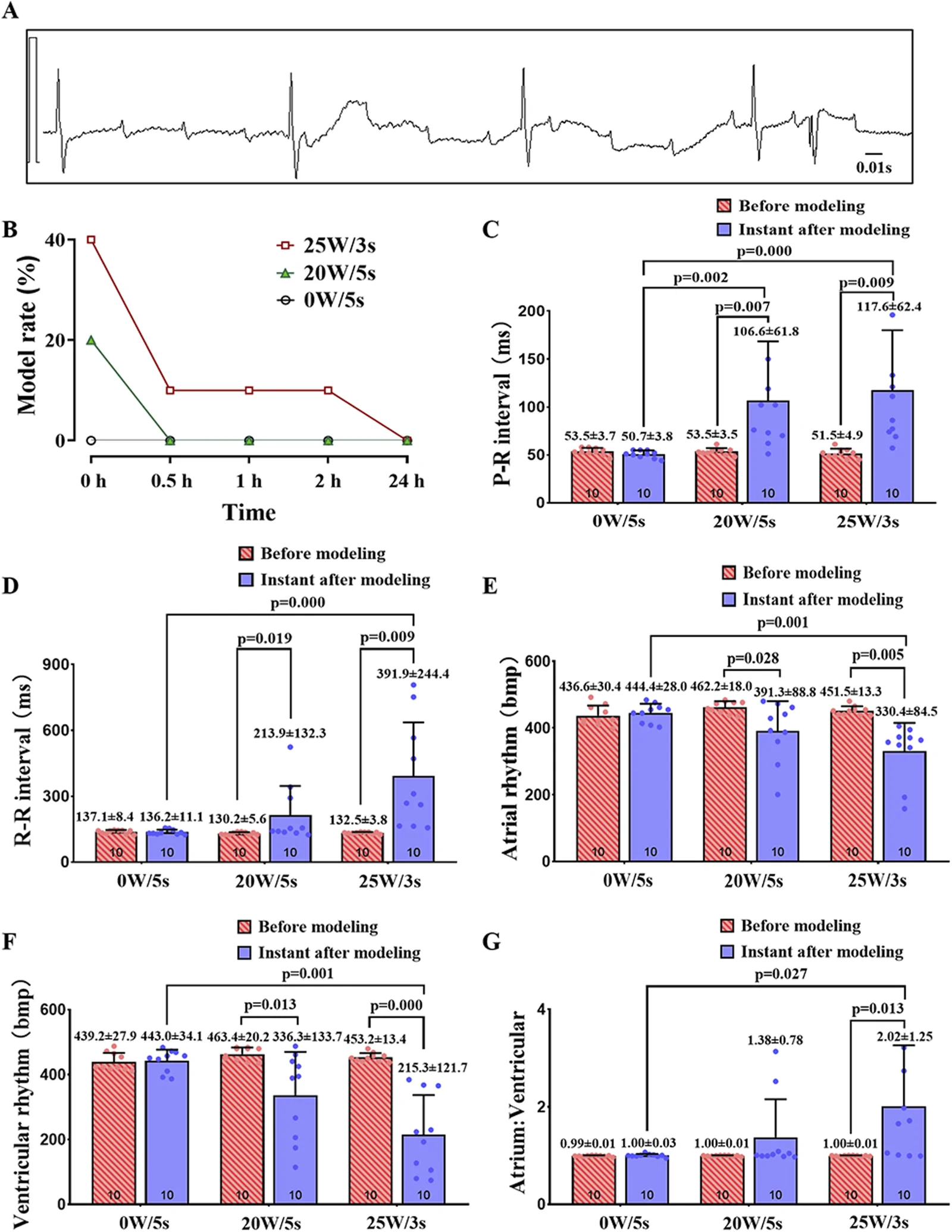
Electrocardiogram (ECG) findings after radiofrequency ablation of the atrioventricular node using an acupuncture needle in rats (n = 10). **(A)** Characteristic complete atrioventricular block (CAVB) ECG showing a ventricular escape rhythm. **(B)** Proportion of rats exhibiting CAVB ECG features. **(C)** P-R interval. **(D)** R-R interval. **(E)** Atrial rhythm. **(F)** Ventricular rhythm. **(G)** Atrial:Ventricular (A:V) beat ratio. Note: The model rate (%) was calculated as follows: (the number of rats exhibiting characteristic CAVB ECG features/the total number of rats) ×100%. All values are presented as the mean ± SD. Paired-samples t-tests or Wilcoxon signed-rank tests was used to assess significant differences in the ECGs results before and after modeling within the same group of rats. One-way ANOVA followed by Tukey’s honestly significant difference (HSD) *post hoc* test and Tamhane’s *post hoc* test or Kruskal–Wallis test was used to evaluate significant differences among groups at the same time point.

Before modeling, normal ECGs with sustained sinus rhythm were observed in all groups (*P* > 0.05). In the 0 W/5 s group, no detectable ECG changes occurred after the AV node tissue was stimulated using the 0 W/5 s ablation setting. In the 20 W/5 s and 25 W/3 s groups, characteristic CAVB ECG features were observed in 20% (2/10) and 40% (4/10) of the rats, respectively. After ablation, the P-R interval became irregular and dissociated, while the R-R interval was significantly prolonged (*P* < 0.05, *P* < 0.01). All six rats exhibiting these features died within 24 h after ablation. Notably, among the 20 rats in the two ablation groups, 11 rats, including four that exhibited CAVB ECG features during ablation, died within 1 h because of extensive hemorrhage caused by adhesion and traction between the needle and myocardial tissue at the ablation site (Figure 4).

#### 3.2.2 A syringe needle induced CAVB ECG features but also caused bleeding and death in rats because of needle adhesion to the tissue

**FIGURE 5:**
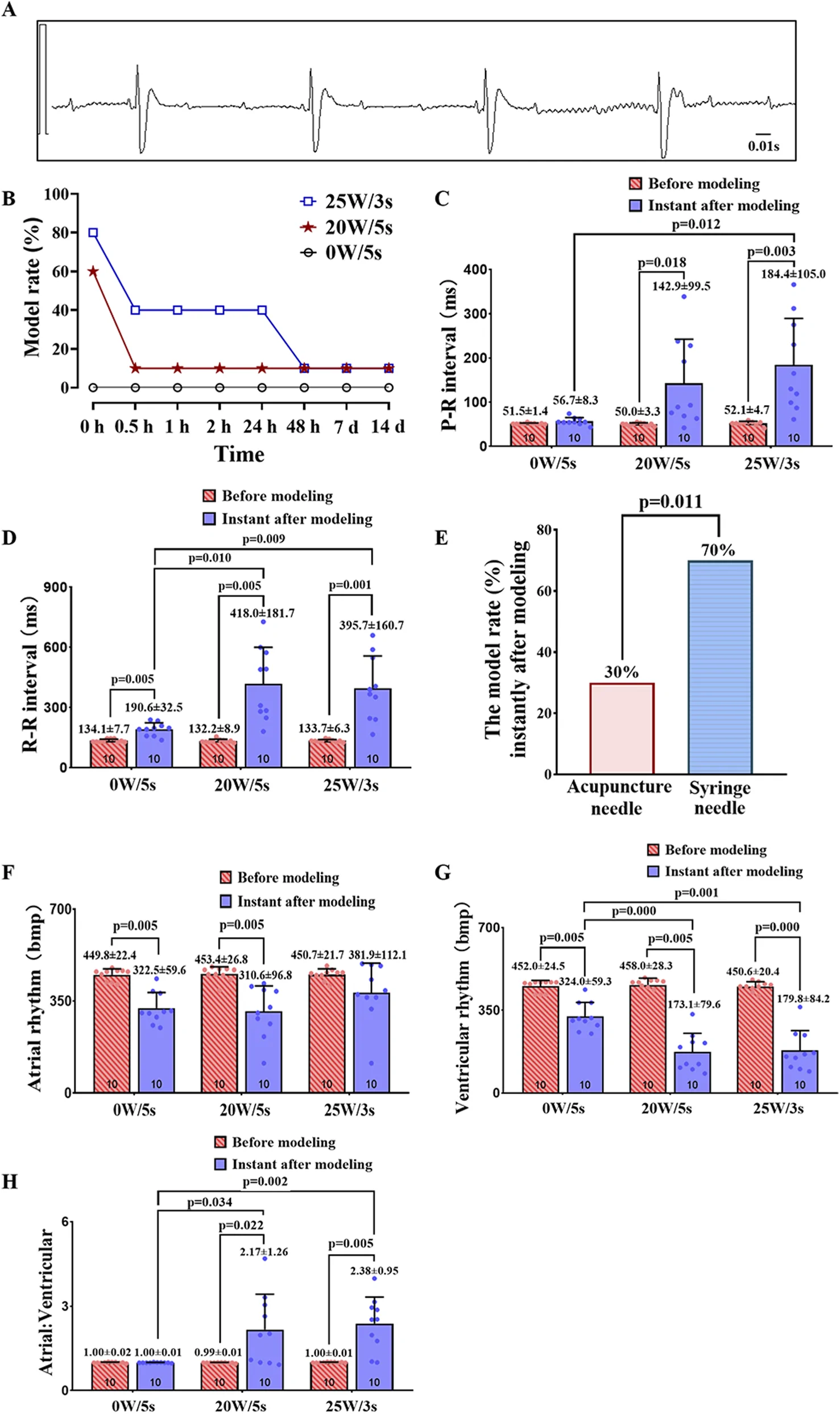
Electrocardiogram (ECG) findings after radiofrequency ablation of the atrioventricular node using a syringe needle in rats (n = 10). **(A)** Characteristic complete atrioventricular block (CAVB) ECG showing a ventricular escape rhythm. **(B)** Proportion of rats exhibiting CAVB ECG features. **(C)** P-R interval. **(D)** R-R interval. **(E)** CAVB model rate (%) immediately after modeling (0 h) in the syringe needle and acupuncture needle groups. **(F)** Atrial rhythm. **(G)** Ventricular rhythm. **(H)** Atrial:Ventricular (A:V) beat ratio. Note: The model rate (%) was calculated as follows: (the number of rats exhibiting characteristic CAVB ECG features/the total number of rats) ×100%. All values are presented as the mean ± SD. Paired-samples t-tests or Wilcoxon signed-rank tests was used to assess significant differences in the ECGs results before and after modeling within the same group of rats. One-way ANOVA followed by Tukey’s honestly significant difference (HSD) *post hoc* test and Tamhane’s *post hoc* test or Kruskal–Wallis test was used to evaluate significant differences among groups at the same time point. Pearson’s chi-squared test was applied to compare rates among groups. A *P* value <0.05 was considered statistically significant.

Before modeling, normal ECGs with sustained sinus rhythm were observed in all rat groups (*P* > 0.05). In the 0 W/5 s group, characteristic CAVB ECG features were not observed after stimulation of the AV node tissue using the 0 W/5 s ablation setting. Although the R-R interval was significantly prolonged (*P* < 0.01), it returned to normal within minutes. Immediately after ablation (0 h), 70% (14/20) of the rats in the syringe needle groups (20 W/5 s and 25 W/3 s groups) exhibited characteristic CAVB ECG features, which was significantly higher than the 30% (6/20) observed in the acupuncture needle groups (*P* < 0.05). Specifically, characteristic CAVB ECG features were observed in 60% (6/10) and 80% (8/10) of the rats in the 20 W/5 s and 25 W/3 s groups, respectively. After ablation, the P-R interval became irregular and dissociated, and the R-R interval was significantly prolonged (*P* < 0.01). Among the 14 rats exhibiting characteristic CAVB ECG features, only two maintained these features for more than 14 days, whereas the other 12 died within 48 h after ablation. Notably, among the 20 rats in the two groups, 13 rats, including 9 that exhibited CAVB ECG features during ablation, died within 1 h because of massive hemorrhage caused by adhesion and traction between the needle and myocardial tissue at the end of ablation (Figure 5).

### 3.3 Myocardial surface radiofrequency ablation successfully established a CAVB rat model

#### 3.3.1 A thick blunt tip induced CAVB ECG features but readily caused acute bleeding and death in rats because of contact between the blunt tip and the aorta

**FIGURE 6:**
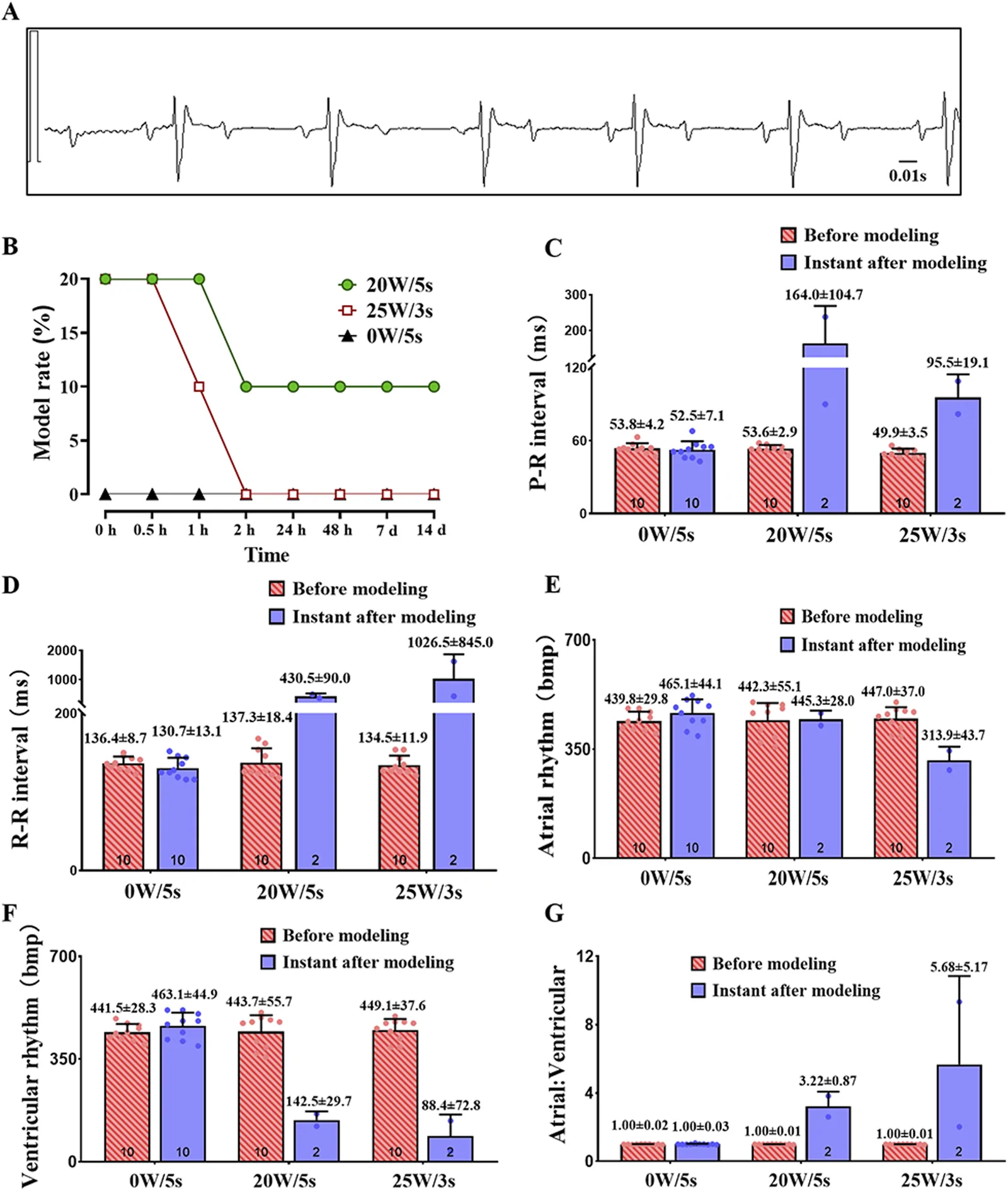
Electrocardiogram (ECG) findings after radiofrequency ablation of the atrioventricular node using a thick blunt tip in rats (n = 2–10). After ablation, only n = 2 rats were evaluated in each of the 20 W/5 s and 25 W/3 s groups because ECGs immediately after modeling (0 h) could not be recorded for animals that died during or immediately after ablation. **(A)** Characteristic complete atrioventricular block (CAVB) ECG showing a ventricular escape rhythm. **(B)** Proportion of rats exhibiting CAVB ECG features. **(C)** P-R interval. **(D)** R-R interval. **(E)** Atrial rhythm. **(F)** Ventricular rhythm. **(G)** Atrial:Ventricular (A:V) beat ratio. Note: The model rate (%) was calculated as follows: (the number of rats exhibiting characteristic CAVB ECG features/the total number of rats) ×100%. All values are presented as the mean ± SD. A paired-samples t-test was used to assess significant differences in the ECGs results before and after modeling in the 0 W/5 s group. HSD *post hoc* test and Tamhane’s *post hoc* test was used to evaluate significant differences among groups before modeling. For the other groups were not performed statistical tests.

Before modeling, all rat groups exhibited normal ECGs with sustained sinus rhythm (*P* > 0.05). In the 0 W/5 s group, no notable ECG changes occurred after the AV node tissue was stimulated using the 0 W/5 s ablation setting. Immediately after ablation, characteristic CAVB ECG features were observed in 20% (2/10) of the rats in each of the 20 W/5 s and 25 W/3 s groups. The P-R interval became irregular and dissociated, and the R-R interval was prolonged. Among the four rats exhibiting characteristic CAVB ECG features, only 1 rat in the 20 W/5 s group maintained these features for more than 14 days, whereas the other 3 rats died within 2 h after ablation. In addition, 80% (16/20) of the rats in these groups died from acute massive hemorrhage caused by inadvertent contact between the thick blunt tip and the aorta during or immediately after ablation. The remaining 20% (4/20) of the rats, in which accidental contact with the aorta did not occur, exhibited characteristic CAVB ECG features after ablation (Figure 6).

#### 3.3.2 A thin blunt tip established a CAVB rat model with a high success rate and good stability

**FIGURE 7:**
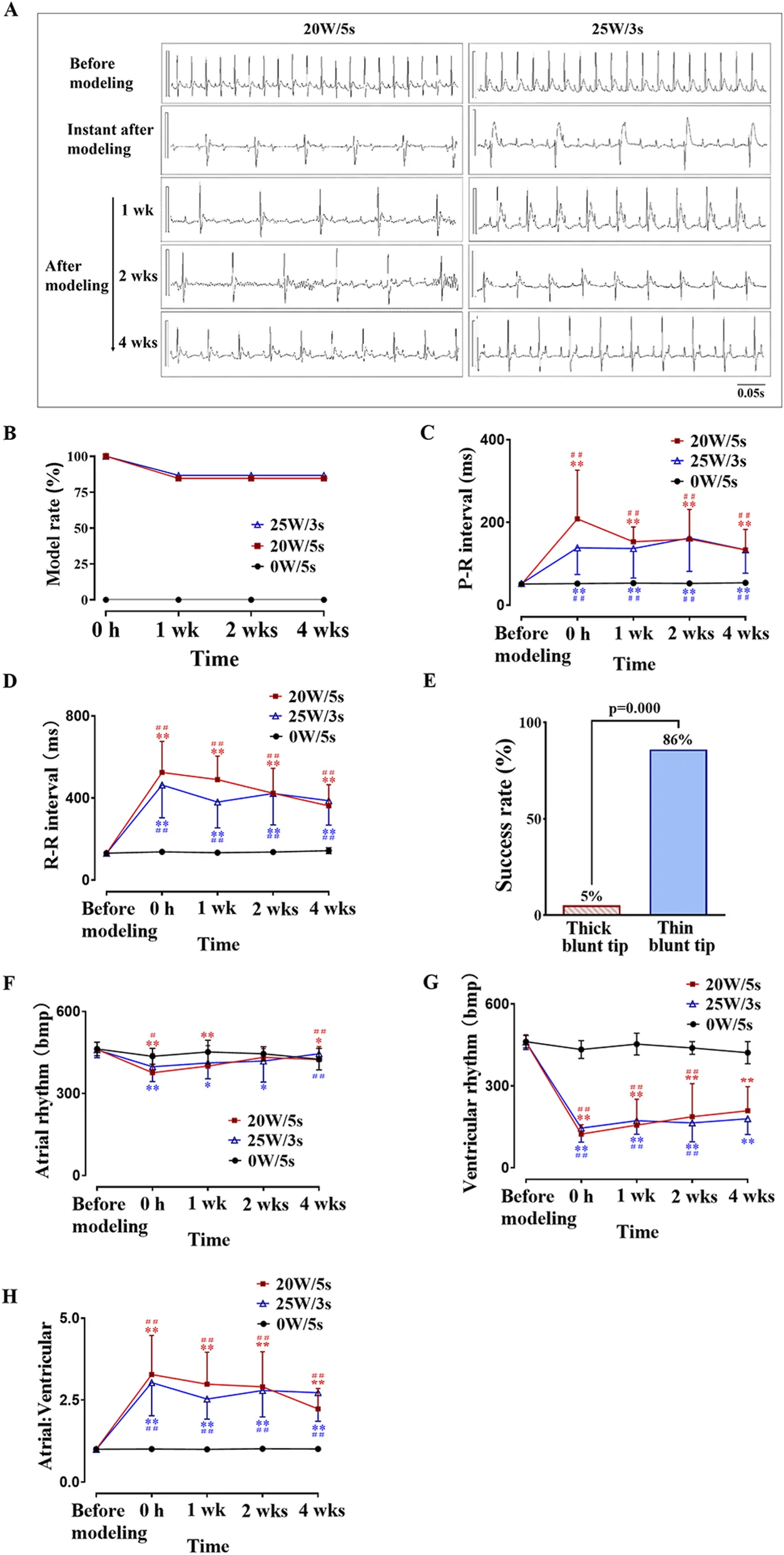
Electrocardiogram (ECG) findings after radiofrequency ablation of the atrioventricular node using a thin blunt tip in rats (n = 10–15). **(A)** Characteristic complete atrioventricular block (CAVB) ECG showing a ventricular escape rhythm. **(B)** Proportion of rats exhibiting CAVB ECG features. **(C)** P-R interval. **(D)** R-R interval. **(E)** Success rates in the thick blunt tip and thin blunt tip groups. **(F)** Atrial rhythm. **(G)** Ventricular rhythm. **(H)** Atrial:Ventricular (A:V) beat ratio. Note: The model rate (%) was calculated as follows: (the number of rats exhibiting characteristic CAVB ECG features/the total number of rats) ×100%. **P < 0.01 compared with the value before modeling. At the same time point, ^##^ *P* < 0.01 compared with the 0 W/5 s group. All values are presented as the mean ± SD. Paired-samples t-tests or Wilcoxon signed-rank tests was used to assess significant differences in the ECGs results before and after modeling within the same group of rats. One-way ANOVA followed by Tukey’s honestly significant difference (HSD) *post hoc* test and Tamhane’s *post hoc* test or Kruskal–Wallis test was used to evaluate significant differences among groups at the same time point. Pearson’s chi-squared test was applied to compare rates among groups. A *P* value <0.05 was considered statistically significant.

Before modeling, all rat groups exhibited normal ECGs with sustained sinus rhythm (*P* > 0.05). In the 0 W/5 s group, no notable ECG changes were detected after the AV node tissue was stimulated using the 0 W/5 s ablation setting. Immediately after ablation, 100% of the rats in both the 20 W/5 s and 25 W/3 s groups exhibited characteristic CAVB ECG features. The P-R interval became irregular and dissociated, the R-R interval was significantly prolonged, and the atrial-to-ventricular (A:V) beat ratio was significantly increased (*P* < 0.01). Moreover, 85% (11/13) of the rats in the 20 W/5 s group and 87% (13/15) of those in the 25 W/3 s group maintained characteristic CAVB ECG features for more than 4 weeks, with no significant difference between the groups (*P* > 0.05). Of the remaining rats, 3 died within 2 h after ablation, while the characteristic CAVB ECG features disappeared in 1 rat within 1 h after ablation. The success rate in the thin blunt tip group was 86% (24/28), which was significantly higher than the 5% (1/20) observed in the thick blunt tip group (*P* < 0.01). (Figure 7).

**FIGURE 8:**
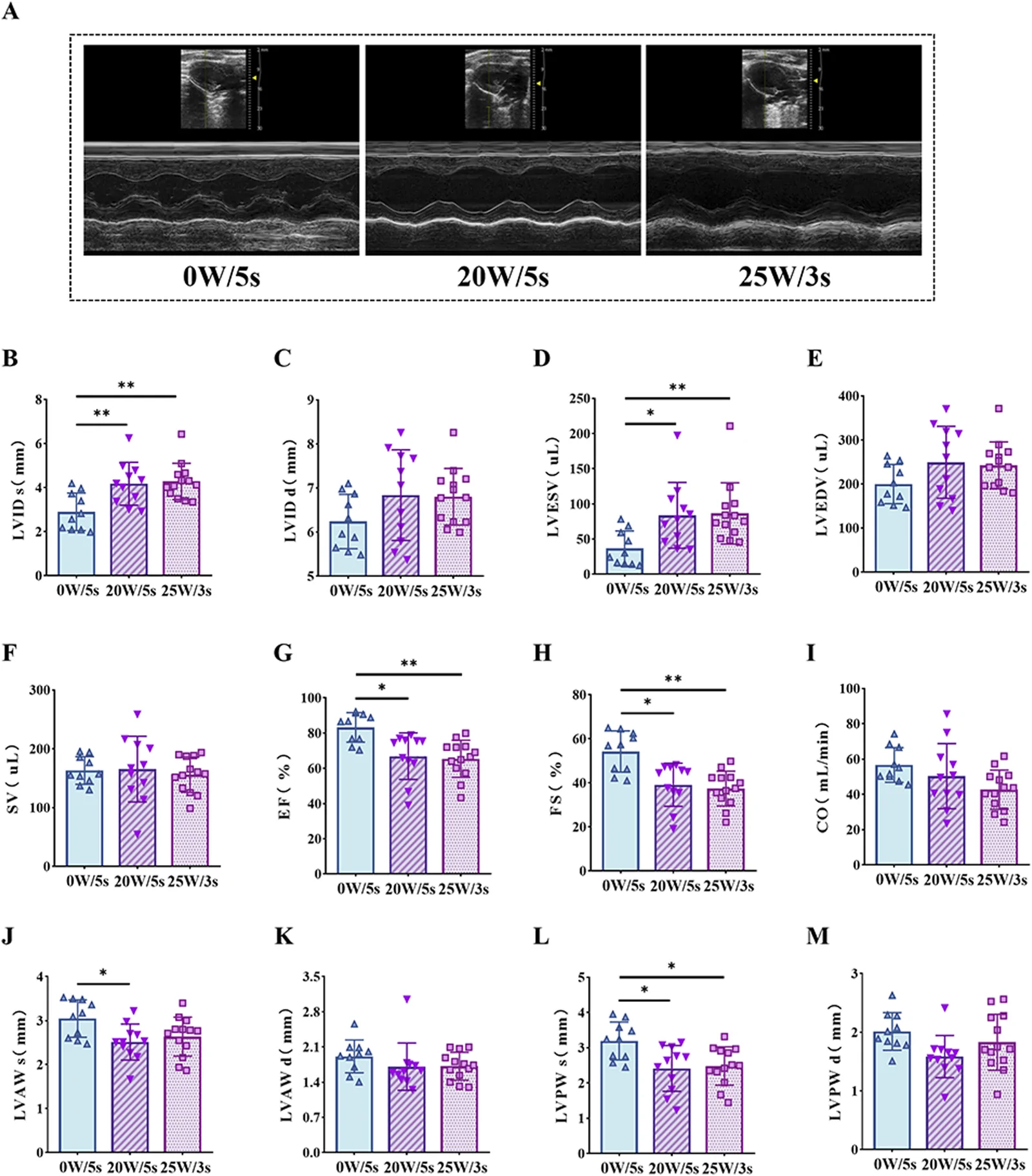
Echocardiographic findings at 4 weeks after radiofrequency ablation using a thin blunt tip in rats (n = 10–13). **(A)** Representative echocardiographic images from each group. **(B–M)** Statistical scatter plots of LVIDs, LVIDd, LVESV, LVEDV, SV, EF, FS, CO, LVAWs, LVAWd, LVPWs, and LVPWd, respectively, in the different groups. Data are presented as the mean ± SD. *P < 0.05, **P < 0.01. One-way ANOVA followed by Tukey’s honestly significant difference (HSD) *post hoc* test and Tamhane’s *post hoc* test or Kruskal–Wallis test was used to assess significant differences among groups.

**FIGURE 9:**
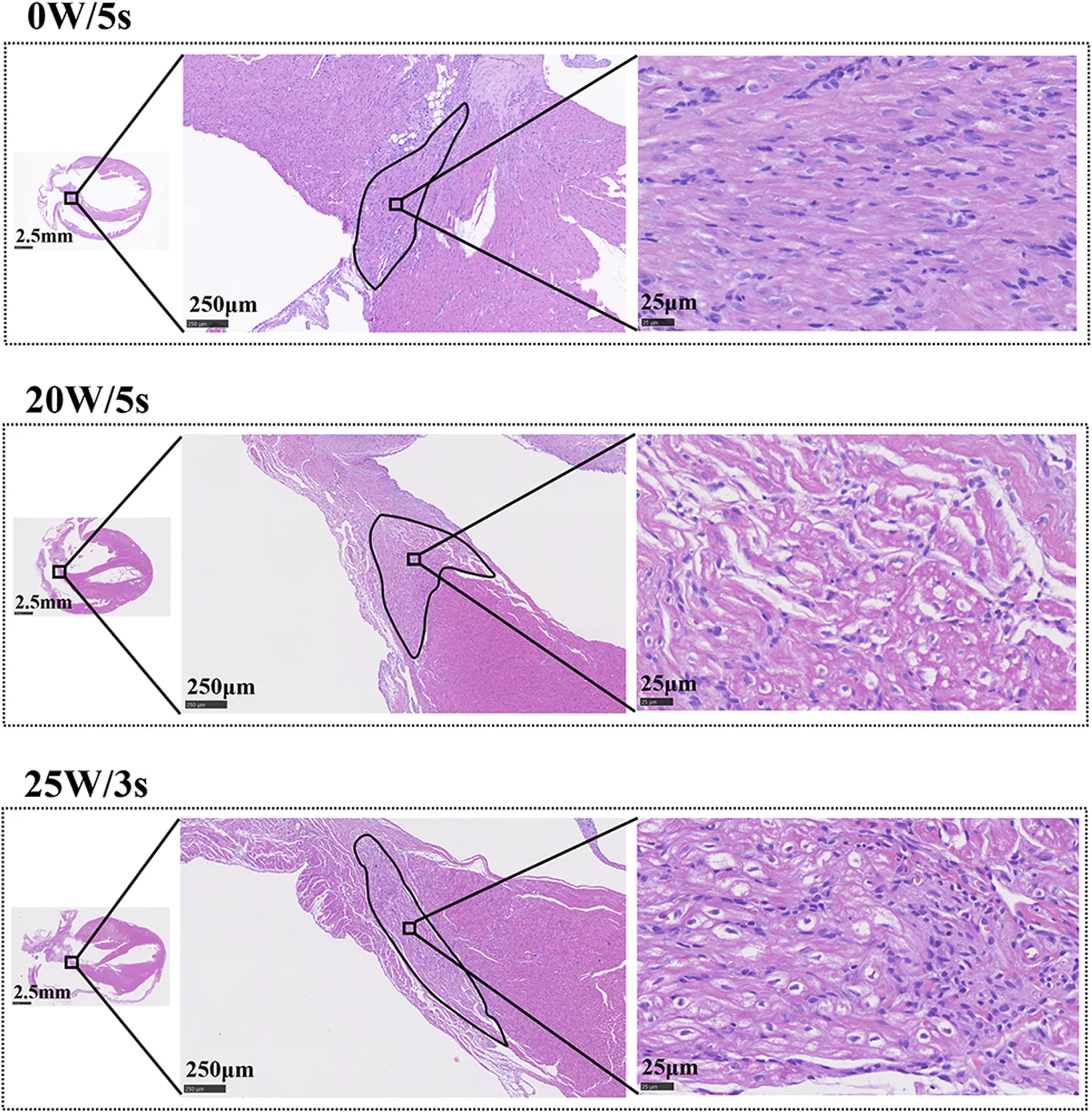
H&E staining of the atrioventricular node at 4 weeks after radiofrequency ablation using a thin blunt tip in rats (n = 5).

**FIGURE 10:**
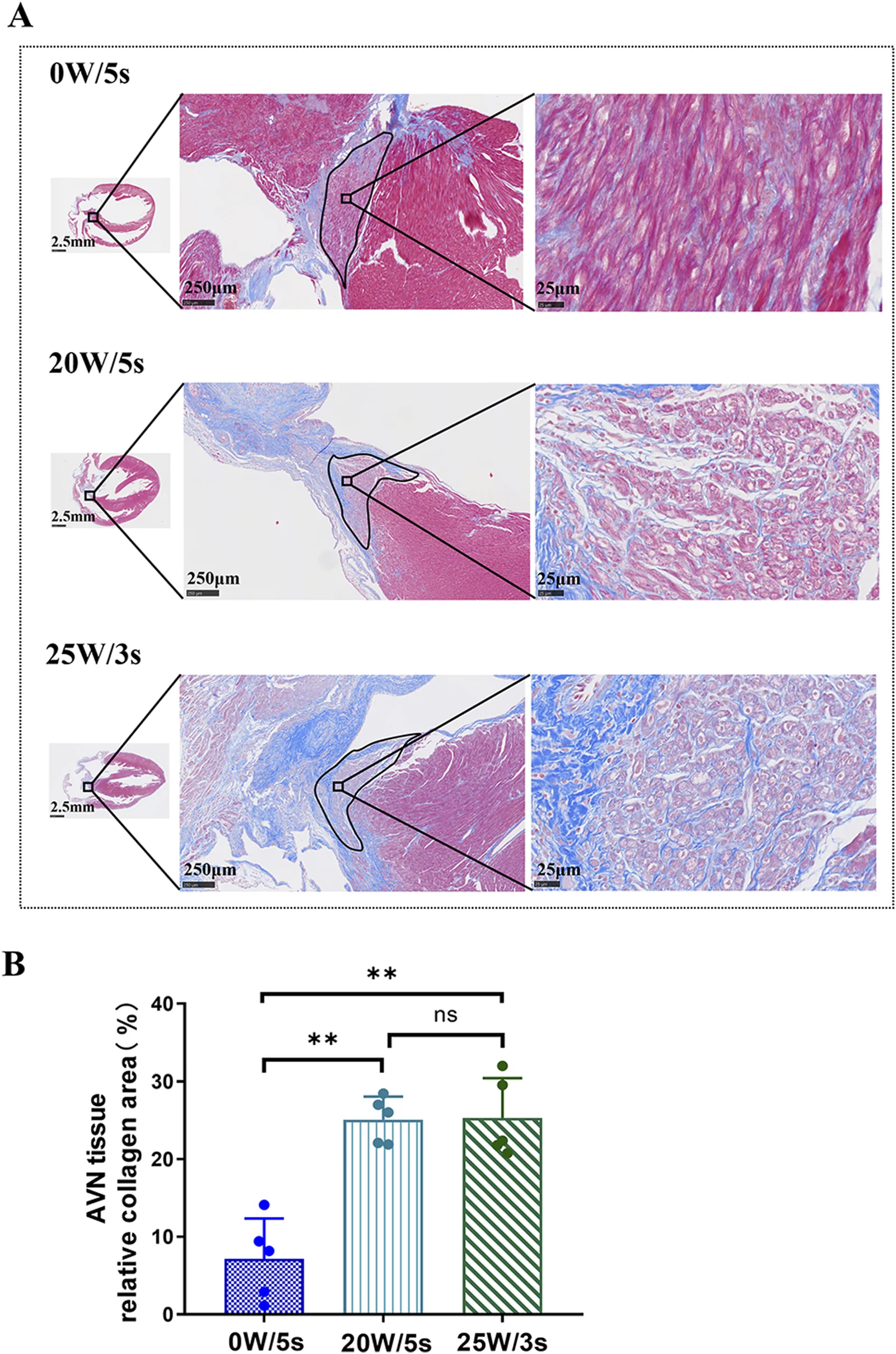
Masson staining of the atrioventricular node at 4 weeks after radiofrequency ablation using a thin blunt tip in rats (n = 5). **(A)** Representative image. **(B)** Relative collagen area (%) in the atrioventricular node tissue. Data are presented as the mean ± SD (n = 5). **P < 0.01. Tukey’s honestly significant difference (HSD) *post hoc* test was used to assess significant differences among groups.

Echocardiography was performed 4 weeks after surgery (Figure 8). Compared with the 0 W/5 s group, LVIDs and LVESV were significantly increased in the 20 W/5 s and 25 W/3 s groups, whereas EF, FS, and LVPWs were significantly decreased (*P* < 0.05 or *P* < 0.01). No statistically significant differences in these parameters were detected between the 20 W/5 s and 25 W/3 s groups *(P* > 0.05). H&E staining showed that the AV node cells in the 0 W/5 s group were compactly and regularly arranged, with small, densely packed cells, normal nuclei, and no evidence of degeneration or necrosis. By contrast, disorganization and fragmentation of the AV node cells, accompanied by substantial inflammatory cell infiltration, were observed in the 20 W/5 s and 25 W/3 s groups (Figure 9). Masson’s trichrome staining revealed minimal blue-stained collagen fibers in the 0 W/5 s group, whereas the relative collagen area was significantly increased in the 20 W/5 s and 25 W/3 s groups (*P* < 0.01). (Figure 10).

## 4 Discussion

CAVB is one of the indications for permanent pacemaker implantation (Fedorowski et al., 2023). At present, CAVB models are predominantly established in large animals, including dogs, pigs, and sheep. Among these animals, canine models of CAVB have been extensively investigated. Radiofrequency ablation has a success rate of 42% to 100%, and the resulting models remain stable for 6–12 weeks; however, prior pacemaker implantation is generally required to improve animal survival after model establishment. Consequently, establishing canine CAVB models is difficult in conventional laboratories because of the procedural complexity, high technical requirements, and considerable expense, with the breeding and testing of one experimental dog costing approximately $4,500 (Loen et al., 2022).

Given the high cost of large-animal models, rats offer advantages because of their small size and relatively low cost. However, experimental studies establishing CAVB models in rats have been relatively scarce over the past 50 years. A PubMed search using the keywords “complete atrioventricular block” and “rat” identified only 9 experimental modeling studies published during this period (Table 2). The modeling methods used in these 9 studies included transvenous electrocautery, chemical ablation, and invasive radiofrequency ablation with an acupuncture needle. Transvenous electrocautery is technically complex, requires advanced procedural skills, and has a low success rate. Furthermore, the remaining reports provide insufficient information regarding the modeling success rate, model maintenance period, or other evaluation parameters. Therefore, accurately assessing the feasibility of chemical and radiofrequency ablation methods remains challenging.

**TABLE 2:** Studies establishing the complete atrioventricular block model in rats from 1972 to 2025.

Method	Open chest or not	Whether to implant a pacemaker	Operation	Results	References
Transvenous electrocautery	NO	NO	The ablation catheter probe was positioned on the surface of the AVN *via* the right jugular vein, and the metal probe was then heated with an electric current, burning only the AV node tissue in contact with it.	Undescribed.	(Alabaster, 1972)
Transvenous electrocautery	NO	NO	Same as reference.	21.6% (37/171) maintained for more than 1 month, 32.2% (55/171) died and 46.2% (79/171) recovered conduction.	(Walford et al., 1988)
Chemical ablation with ethanol	YES	NO	The 70% ethanol solution directly injected into the AVN.	82.1% (23/28) rats showed CAVB ECG characteristics immediately after ablation. Among them, 17.4% (4/23) maintained for 7 days, and the outcome of the remaining rats was not described.	(Lee et al., 1998)
Chemical ablation with ethanol	YES	NO	Same as reference.	15 rats were maintained for more than 14 days, but the total number was not described.	(Yokokawa et al., 2008)
Electrocautery ablation with acupuncture needle	YES	NO	The acupuncture needle is invaded into the AVN, and then the electric current heats the acupuncture needle to burn the AVN tissue in contact with it.	43% (18/42) maintained for more than 15 days, 38% (16/42) maintained for more than 30 days, and the remaining 19% (8/42) resumed conduction.	(da Cunha et al., 2014)
Electrosurgical ablation with acupuncture needle	YES	NO	The acupuncture needles delivered high-frequency electrical energy to the contact points of the AVN tissue electrodes and the area around the contact points for ablation.	71% (15/21) of 6-month-old female rats maintained for more than 4 weeks. And all males and 3-month-old females died within 3 days of modeling.	(Kim et al., 2019)
Electrocautery ablation with acupuncture needle	YES	NO	Same as reference.	63% (28/44) showed CAVB ECG characteristics immediately after ablation, but the duration of maintenance was not described.	(Ali et al., 2020)
Mechanical damage	YES	NO	Mechanical damage induced by cutting high interventricular septum.	Undescribed.	(Sanchez et al., 2022)
Radiofrequency ablation with needle	YES	NO	Performed by advancing the distal tip of a sharp needle into the AVN region by 2–3 mm and passing electrical power through the proximal tip of the needle.	25% (6/24) survived at 4 weeks after subepicardial CAVB surgery. 77% (20/26) survived at 4 weeks after epicardial CAVB surgery.	(Weltz et al., 2025)

First, the establishment of an animal model of CAVB requires accurate identification of the AV node location. In 1998, Lee et al. (1998) first precisely described the anatomical location of the AV node in rats and identified a white fat pad between the aortic root and the medial wall of the right auricle. Under physiological conditions, however, the right auricle of a rat completely covers this fat pad and obstructs the operator’s view. During preliminary experiments, fixation of the right auricle using surgical forceps or sutures resulted in bleeding and loosening. Therefore, sterile cotton swabs were ultimately selected to retract the right auricle and expose the fat pad.

First, this study attempted to destroy the AV node tissue in rats using ethanol. However, ethanol ablation had a low success rate and did not produce a stable CAVB rat model. Formaldehyde ablation was subsequently evaluated as an alternative modeling method. However, only 1 rat maintained characteristic CAVB ECG features for more than 14 days after ablation. Therefore, neither ethanol nor formaldehyde ablation successfully established a stable CAVB rat model. These findings suggest that the characteristic CAVB ECG features induced by ethanol ablation were transient and readily resolved, whereas formaldehyde ablation was associated with increased mortality in rats. Recovery after ethanol ablation may have been related to the limited and uneven injury produced in the AV node tissue despite injection of a high dose of ethanol solution. Moreover, injection of high-dose formaldehyde may cause extensive myocardial injury, induce thrombosis within the ventricular cavity, and ultimately lead to animal death.

A rat model of CAVB was subsequently established by attempting to destroy the AV node tissue through myocardial invasive radiofrequency ablation using an acupuncture needle. The findings indicated that this method did not effectively establish a CAVB rat model because all affected rats died within 24 h after modeling because of massive hemorrhage. The small number of rats exhibiting characteristic CAVB ECG features immediately after AV node ablation may be attributable to the limited diameter of the acupuncture needle, which provided a small contact area with the AV node and consequently produced small and mild ablation lesions. Therefore, the acupuncture needle was replaced with a 1 mL syringe needle, which had a larger contact tip. However, only two rats maintained characteristic CAVB ECG features for more than 14 days, whereas the other 12 died within 48 h after ablation. During model establishment, adhesion and traction between the needle tip and myocardial tissue caused substantial hemorrhage, resulting in animal death within 1 hour. Thus, a CAVB modeling method that could prevent hemorrhage during withdrawal of the needle was required.

Because the invasive radiofrequency ablation methods described above caused tissue adhesion and severe bleeding, the feasibility of directly injuring the AV node from the myocardial surface without penetrating the myocardial tissue was investigated. Myocardial surface radiofrequency ablation was therefore performed by carefully placing the thick blunt tip of the high-frequency electrosurgical knife on the myocardial surface between the fat pad and the aorta. The results showed that myocardial surface radiofrequency ablation using a thick blunt tip had a low success rate. Although characteristic CAVB ECG features and a ventricular escape rhythm could be induced after ablation, most animals died from acute hemorrhagic shock because the thick blunt tip contacted the aorta during the procedure. To reduce animal mortality, the size of the blunt tip used for myocardial surface ablation had to be decreased to prevent accidental contact with the aorta and the resulting bleeding. In this study, a thin blunt tip comprising a smooth, rounded 0.8 mm wire connected to a high-frequency electrosurgical knife was used for radiofrequency ablation of the AV node in rats. The findings showed that this method successfully established a CAVB rat model, although 3 rats still died during the observation period. These deaths may have been caused by CAVB itself. Pacemaker implantation may therefore further improve rat survival. This finding is consistent with that reported by Weltz et al., who demonstrated that the 4-week survival rate after myocardial surface radiofrequency ablation (77%) was significantly higher than that after myocardial invasive radiofrequency ablation (25%) (Weltz et al., 2025). These results support myocardial surface radiofrequency ablation as the preferred approach for establishing a rat CAVB model.

In addition, cardiac ultrasonography and histopathological examinations were performed to investigate structural and functional changes in the left ventricle after radiofrequency ablation using a thin blunt tip. At 4 weeks after modeling, cardiac function was significantly impaired in the CAVB rats, and degeneration, necrosis, and extensive fibrosis were observed in the AVN region. These findings further indicate that myocardial surface radiofrequency ablation using a thin blunt tip is the preferred approach for establishing a CAVB rat model. Moreover, the two myocardial surface ablation settings produced similar modeling outcomes. This finding suggests that the ablation parameters can be adjusted flexibly according to individual differences among rats, variations in laboratory conditions, and the instruments used. The general principle is that lower power requires a longer ablation duration, whereas higher power permits a shorter ablation duration.

Nevertheless, this study has several limitations. First, the AV node was localized anatomically according to previously published findings. Although this method is simple and convenient, it may not precisely identify the location of the AV node. Local electrophysiological indicators could be incorporated in future studies to achieve more accurate localization of the AV node. Second, long-term monitoring of ECG features using telemetry could provide stronger evidence regarding model stability. These aspects will be further investigated, and the modeling method will be continuously optimized in subsequent studies.

## 5 Conclusion

Chemical ablation, myocardial invasive radiofrequency ablation, and myocardial surface radiofrequency ablation using a thick blunt tip did not achieve satisfactory outcomes when establishing the CAVB rat model. By contrast, myocardial surface radiofrequency ablation using a thin blunt tip provided a simple and reliable method for establishing a stable CAVB rat model with a high success rate. Three important factors should be considered during model establishment: 1) the probe surface should be smooth to prevent mechanical myocardial injury; 2) the probe should be appropriately sized to prevent excessive contact with the aorta while avoiding inadequate ablation caused by an excessively small tip; and 3) the ablation parameters should be adjusted flexibly according to the principle that higher power requires a shorter ablation duration, whereas lower power requires a longer ablation duration.

## Data Availability

The raw data supporting the conclusions of this article will be made available by the authors, without undue reservation.

## Supplementary material

SUPPLEMENTARY VIDEO 1 Electrocardiographic features of complete atrioventricular block, including a ventricular escape rhythm, observed in rats immediately after modeling.

## References

[B1] Alabaster C. T. (1972). A method of producing permanent, complete atrioventricular block in the rat. Br. J. Pharmacol. 46, 581P. 4656653 PMC1666533

[B2] Ali A. Redfors B. Lundgren J. Alkhoury J. Oras J. Gan L.-M. (2020). The importance of heart rate in isoprenaline-induced takotsubo-like cardiac dysfunction in rats. Esc. Heart Fail 7, 2690–2699. 10.1002/ehf2.12858 32686334 PMC7524126

[B3] Bru P. Lauribe P. Rouane A. Nadi M. Prieur G. Ricard P. (2002). Catheter ablation using very high frequency current: effects on the atrioventricular junction and ventricular myocardium in sheep. Europace 4, 69–75. 10.1053/eupc.2001.0205 11846319

[B4] da Cunha D. N. Q. Pereira V. G. Favarato L. S. C. Okano B. S. Daibert A. P. F. Monteiro B. S. (2014). Acute and chronic observations of complete atrioventricular block in rats. Lab. Anim. 48, 237–249. 10.1177/0023677214530905 24759570

[B5] Fedorowski A. Rosengren P. Pirouzifard M. Sundquist J. Sundquist K. Sutton R. (2023). Familial associations of complete atrioventricular block: a national family study in Sweden. Circ Genomic Precis. Med. 16, e003654. 10.1161/CIRCGEN.121.003654 36802810

[B6] Huang S. K. Bharati S. Graham A. R. Lev M. Marcus F. I. Odell R. C. (1987). Closed chest catheter desiccation of the atrioventricular junction using radiofrequency energy--a new method of catheter ablation. J. Am. Coll. Cardiol. 9, 349–358. 10.1016/s0735-1097(87)80388-1 3805526

[B7] Joury A. Bob-Manuel T. Sanchez A. Srinithya F. Sleem A. Nasir A. (2021). Leadless and wireless cardiac devices: the next frontier in remote patient monitoring. Curr. Probl. Cardiol. 46, 100800. 10.1016/j.cpcardiol.2021.100800 33545511

[B8] Kim N. K. Wolfson D. Fernandez N. Shin M. Cho H. C. (2019). A rat model of complete atrioventricular block recapitulates clinical indices of bradycardia and provides a platform to test disease-modifying therapies. Sci. Rep. 9, 6930. 10.1038/s41598-019-43300-9 31061413 PMC6502940

[B9] Lee R. J. Sievers R. E. Gallinghouse G. J. Ursell P. C. (1998). Development of a model of complete heart block in rats. J. Appl. Physiol. (1985) 85, 758–763. 10.1152/jappl.1998.85.2.758 9688757

[B10] Lehmann H. I. Deisher A. J. Takami M. Kruse J. J. Song L. Anderson S. E. (2017). External arrhythmia ablation using photon beams: ablation of the atrioventricular junction in an intact animal model. Circ. Arrhythm. Electrophysiol. 10, e004304. 10.1161/CIRCEP.116.004304 28408649

[B11] Loen V. Vos M. A. van der Heyden M. A. G. (2022). The canine chronic atrioventricular block model in cardiovascular preclinical drug research. Br. J. Pharmacol. 179, 859–881. 10.1111/bph.15436 33684961 PMC9291585

[B12] Sanchez L. Mesquita T. Zhang R. Liao K. Rogers R. Lin Y.-N. (2022). MicroRNA-dependent suppression of biological pacemaker activity induced by TBX18. Cell Rep. Med. 3, 100871. 10.1016/j.xcrm.2022.100871 36543116 PMC9798022

[B13] Sill B. Roy N. Hammer P. E. Triedman J. K. Sigg D. C. Kelly M. F. (2011). Development of an ovine model of pediatric complete heart block. J. Surg. Res. 166, e103–e108. 10.1016/j.jss.2010.11.878 21227467 PMC3073560

[B14] Smoczyńska A. Loen V. Aranda A. Beekman H. D. M. Meine M. Vos M. A. (2020). High-rate pacing guided by short-term variability of repolarization prevents imminent ventricular arrhythmias automatically by an implantable cardioverter-defibrillator in the chronic atrioventricular block dog model. Heart Rhythm. 17, 2078–2085. 10.1016/j.hrthm.2020.07.023 32710972

[B15] Tjong F. V. Y. Reddy V. Y. (2017). Permanent leadless cardiac pacemaker therapy: a comprehensive review. Circulation 135, 1458–1470. 10.1161/CIRCULATIONAHA.116.025037 28396380

[B16] Walford G. D. Spurgeon H. A. Lakatta E. G. (1988). Diminished cardiac hypertrophy and muscle performance in older compared with younger adult rats with chronic atrioventricular block. Circ. Res. 63, 502–511. 10.1161/01.res.63.3.502 2970333

[B17] Weltz A. N. Fortuno-Miranda L. Kolandaivelu A. Fan J. Ramirez R. J. Cho H. C. (2025). A highly effective, rodent model of complete heart block by epicardial radiofrequency ablation. Sci. Rep. 15, 40740. 10.1038/s41598-025-24479-6 41258478 PMC12630668

[B18] Yokokawa M. Ohnishi S. Ishibashi-Ueda H. Obata H. Otani K. Miyahara Y. (2008). Transplantation of mesenchymal stem cells improves atrioventricular conduction in a rat model of complete atrioventricular block. Cell Transpl. 17, 1145–1155. 10.3727/096368908787236594 19181209

